# Quantified versus willful handgrip exercises for the prevention of PICC-related thrombosis: A meta-analysis and systematic review

**DOI:** 10.1097/MD.0000000000032706

**Published:** 2023-03-10

**Authors:** Hongliang Luo, Cheng Jin, Xiaohong Li, Yinzhu Jiang, Jing Zhou

**Affiliations:** a Department of cardiothoracic surgery, Children’s Hospital of Nanjing Medical University, Nanjing, China; b Department of urology surgery, Children’s Hospital of Nanjing Medical University, Nanjing, China; c Department of neonatal surgery, Children’s Hospital of Nanjing Medical University, Nanjing, China.

**Keywords:** care, grip exercises, meta-analysis, nursing, PICC, thrombosis

## Abstract

Thrombosis is a common yet serious complication in patients with peripherally inserted central venous catheter (PICC), the prevention of thrombosis is very important to the prognosis of PICC patients. We aimed to evaluate the effects of quantified versus willful grip exercises for the prevention of PICC-related thrombosis, to provide evidence to the clinical nursing care of PICC patients.

**Methods:**

Two authors searched PubMed et al databases for randomized controlled trials (RCTs) comparing the effects of quantified versus willful grip exercises in PICC patients up to August 31, 2022. Quality assessment and data extraction were independently performed by 2 researchers, and meta-analysis was performed using RevMan 5.3 software.

**Results:**

15 RCTs involving 1741 PICC patients were finally included in this meta-analysis. Synthesized outcomes indicated that compared with willful grip exercises, quantified grip exercises reduced the incidence of PICC-related thrombosis (odds ratio = 0.19, 95% confidence interval [CI]: 0.12–0.31) and infection (odds ratio = 0.30, 95% CI: 0.15–0.60) in PICC patients, increased the maximum venous velocity (mean difference = 3.02, 95% CI: 1.87–4.17) and mean blood flow (mean difference = 3.10, 95%CI: 1.57–4.62) in PICC patients (all *P* < .05). There were no publication biases amongst the synthesized outcomes (all *P* > .05).

**Conclusion:**

Quantified grip exercises can effectively reduce the occurrence of PICC-related thrombosis and infection, improve the venous hemodynamics. Limited by study population and regions, large-sample, and high-quality RCTs are still needed in the future to further evaluate the effects and safety of quantified grip exercises in PICC patients.

## 1. Introduction

Peripherally inserted central venous catheter (PICC) refers to the placement of peripheral veins such as the precious vein, cephalic vein, etc, and the tip of the catheter is positioned at the cavoatrial junction or the middle and lower 1/3 m of the superior vena cava vein near the cavoatrial junction.^[1]^ PICC can be operated by nurses alone, the placement process is simple and safe with high success rate.^[2,3]^ Besides, PICC has the advantages of long use time and easy maintenance. PICC provides a medium- and long-term intravenous treatment access for patients who require long-term infusion of chemotherapy drugs, blood products or intravenous nutrition.^[4]^ However, as an invasive and foreign body operation, long-term indwelling of PICC will also lead to numerous complications. Previous literatures^[5,6]^ have reported that some complications are related to PICC in clinical include phlebitis, PICC-associated bloodstream infection thrombosis, etc. The occurrence of these complications will increase the financial burden of the patients and reduce the quality of life of the patients. If they cannot be corrected in time, it will cause unplanned PICC extubation and lead to treatment interruption.^[7]^ It been reported that symptomatic thrombosis occurs in 0.5% to 4.7% of PICC patients, and asymptomatic thrombosis occurs in up to 50% of PICC patients.^[8]^ PICC patients with thrombosis will be accompanied by symptoms such as upper limb swelling, tenderness around the implantation site, and elevated skin temperature. In severe cases, upper limb dysfunction may also occur.^[9]^ Therefore, the prevention and care of PICC-related thrombosis is of great significance to the prognosis of patients.

The “2021 Practice Standards for Infusion Therapy”^[10]^ has encouraged patients with indwelling PICCs to perform early, normal daily activities, and mild physical exercise, but it has not specify the specific early hand activity program. There are many researches^[11–13]^ on the methods and modes of limb exercise on the side of PICC catheterization worldwide, hand and upper limb exercises are widely used in clinical practice for prevention, but there is no quantitative standard for the duration, frequency, and frequency of exercise. Several previous studies^[14–16]^ have reported the effects of quantified versus willful grip exercises for the prevention of PICC-related thrombosis, yet the results remain inconsistent. Therefore, this meta-analysis and systematic review aimed to investigate the effects of quantified versus willful grip exercises, to provide evidences to the clinical nursing care of PICC patients.

## 2. Methods

Ethical review was not necessary since our study was a meta-analysis and systematic review. This meta-analysis was conducted in accordance to the guidelines for preferred reporting items for systematic reviews and meta-analyses statement.^[17]^

### 2.1. Retrieval strategy

The 2 researchers searched the associated studies by combinations of search terms and keywords. The search terms used in this meta-analysis were as follows: (“fist exercise” OR “ball-holding sport” OR “handgrip exercise” OR “quantitative physical intervention” OR “functional exercise”) AND (“PICC” OR “peripherally inserted central catheter” OR “PICC-related VT” OR “venous thrombosis” OR “catheter-related thrombosis.” Databases searched in this meta-analysis included PubMed, CINAHL and Cochrane Library, China Biomedical Literature Database, China national knowledge infrastructure, Wanfang and Weipu databases. The retrieval limit was from the establishment of the database to August 31, 2022.

### 2.2. Inclusion and exclusion criteria

The inclusion criteria for this meta-analysis were as follows: Study design: randomized controlled trials (RCTs) comparing the effects of quantified versus willful grip exercises in PICC patients; Research population: patients with indwelling PICCs; Interventions: the experimental group underwent quantified grip exercises by designed nursing care plans, the control group underwent grip exercises at their own willingness; Observation indicators: The main indicators are the incidence of venous thrombosis and venous hemodynamics, and the secondary indicators are the infection rate associated to PICC. Venous thrombosis and venous blood flow velocity were examined with Doppler color ultrasound. The maximum venous blood flow velocity refers to the maximum blood flow velocity per second in the blood vessel. The exclusion criteria for this meta-analysis were: Replicated publications; Literature reports for which the full-text could not be obtained or the data could not be extracted.

### 2.3. Literature quality evaluation

The quality of included RCTs was assessed in this meta-analysis according to the Cochrane Risk of Bias Assessment Tool.^[18]^ The tool evaluated following items: random sequence generation, allocation concealment, blinding, completeness of outcome data, selective outcome reporting, and other sources of bias. The evaluation was conducted by 2 researchers independently and reached a consensus through discussion. In case of disagreement, the evaluation was conducted by a third researcher.

### 2.4. Data extraction

We used standardized data collection forms to extract key information. Any discrepancies in the extraction process were resolved by consensus. We also attempted to contact the authors for additional data or to clarify missing details. Two reviewers independently extracted the following information: first author, year of publication, details of targeted population, details of grip exercises, and study outcomes.

### 2.5. Statistical methods

This meta-analysis used RevMan 5.3 software (http://www.cochrane.org/software/revman.htm) for statistical analysis of relevant data. The odds ratio (OR) pooled statistic was used for dichotomous variables, and the mean difference (MD) pooled statistic was used for continuous variables, and 95% confidence interval (CI) were calculated for all statistics. The χ2 test was used to determine whether there was heterogeneity between studies. If there was no significant heterogeneity (*P* ≥ .10, *I*^2^ < 50%), a fixed-effect model was used for meta-analysis. If there was significant heterogeneity (*P* < .10, *I*^2^ ≥ 50%), a random-effects model was used. If *P* < .1 and the source of heterogeneity could not be judged, no meta-analysis was performed, and descriptive analysis was used. Publication bias was analyzed using funnel plots and Egger regression test. In this meta-analysis, *P* < .05 was considered as a statistically significant difference between groups.

## 3. Results

### 3.1. RCTs selection

**Figure 1.**
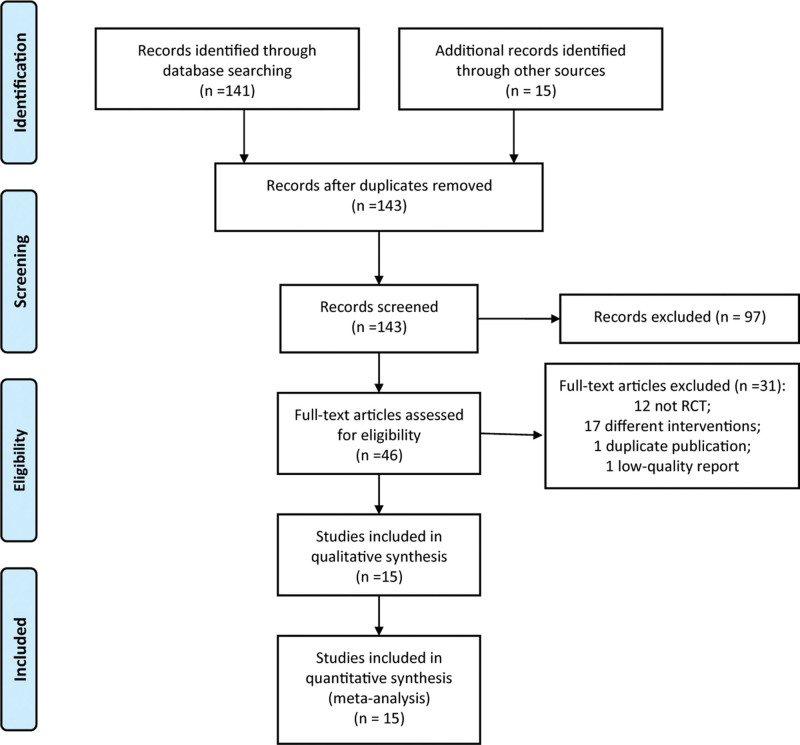
PRISMA flow diagram of RCT selection. PRISMA = preferred reporting items for systematic reviews and meta-analyses, RCT = randomized controlled trial.

A total of 156 literatures were retrieved in the initial search, and 143 literatures were initially screened by the reading title and abstracts. After the full-text reading was carried out to eliminate the literatures that did not meet the inclusion criteria, 15 RCTs^[19–33]^ were finally included in this meta-analysis (Fig. 1).

### 3.2. The characteristics of included RCTs

Of the included 15 RCTs,^[19–33]^ a total of 1741 PICC patients were involved, 897 patients underwent the quantified grip exercises, and 844 patients underwent willful grip exercises. The characteristics of included RCTs are presented in Table 1.

**Table 1:** The characteristics of included RCTs.

	Sample size		Age		Interventions		
RCT	Experimental group	Control group	Experimental group	Control group	Experimental group	Control group	Outcomes
Chen 2016	40	40	55.3 ± 8.1	56.6 ± 9.3	25 times/min, weak grip strength, 2 min each time to make a fist, repeated at 5 min intervals, 0.5 h, 4 times/d	Grip exercises based on patients willingness	①, ②, ④, ⑨
Hu 2017	179	127	60.8 ± 9.4	59.3 ± 8.9	Grip the ball to reduce the diameter by about 1/2, the time ratio of clenching and releasing fists is 1:1, the frequency is 20–25 times/min, each clenching fist is 2 minutes, resting for 5 minutes, lasting 30 minutes, 5–6 sets/d	Grip exercises based on patients willingness	①, ⑧, ⑨
Hui 2018	43	43	55.9 ± 6.8	58.5 ± 8.1	Pinching the ball diameter to 1/2, hold tightly for 10 s, relax for 10 s, 25–30 times/group in a row, 3 groups/d	Grip exercises based on patients willingness	①, ②, ③, ⑤
Lin 2019	56	56	61.1 ± 10.3	60.2 ± 9.9	Grip the ball to reduce the diameter to 1/2 of the normal size, hold for 3 s and then release for 3 s, 10–30 times/min, 30 min/ group, 5 groups/d.	Grip exercises based on patients willingness	①, ⑤, ⑧
Lu 2021	50	52	71.4 ± 10.6	68.5 ± 9.2	Grip the ball as hard as possible, hold the ball for 10 s each time, then relax for 10 s, 10 minutes/set,3 sets/d.	Grip exercises based on patients willingness	①, ⑥, ⑧
Ren 2018	52	52	60.8 ± 9.5	58.2 ± 8.4	Firmly grip the ball for 10 s, then relax for 10 s, repeat 20 times/set, 3 sets/d.	Grip exercises based on patients willingness	①, ②, ⑥, ⑦
Wang 2016	42	42	51.4 ± 9.4	52.1 ± 10.7	Hold the ball for 10 s, rest 10 s, hold 20 times/set, 3 sets/d.	Grip exercises based on patients willingness	①, ②, ③
Wang 2017	88	87	54.1 ± 8.6	55.2 ± 8.4	Grip the ball to 1/2 of the normal size, hold for 3 s and then relax for 3 s, 10–30 times/min, hold the ball for 2 minutes each time, rest for 5 minutes in between, the duration is about 30 minutes, 5 sets/d.	Grip exercises based on patients willingness	①, ⑤, ⑧
Xin 2019	19	17	66.3 ± 8.4	67.6 ± 9.1	Weak grip strength, 25 reps/min, combined with wrist and elbow movements for 2 minutes.	Grip exercises based on patients willingness	①, ②, ③, ④
Xun 2020	30	30	51.6 ± 9.2	52.4 ± 10.2	Hold the ball for 15 s, rest 5 s, hold 20 times/set, 5 sets/d.	Grip exercises based on patients willingness	①, ④, ⑤
Yin 2017	60	60	54.9 ± 9.7	55.3 ± 9.8	Slowly and firmly clenched the fist, flexed and extended the knuckles, wrist, elbow, held for 3 s and then relaxed, repeated 30 times, 3 sets/d.	Grip exercises based on patients willingness	①, ⑤, ⑥, ⑦
Yin 2017	60	60	53.94 ± 9.13	54.02 ± 10.44	Slowly and firmly clenched the fist, flexed and extended the knuckles, wrist, elbow, held for 3 s and then relaxed, repeated 30 times, 3 sets/d.	Grip exercises based on patients willingness	①, ⑤, ⑥, ⑦
Zheng 2019	53	53	62.1 ± 8.6	60.2 ± 9.5	Hold the ball for 5 s, release for 5 s, 20 reps/set, 6 sets/d.	Grip exercises based on patients willingness	①, ②, ③
Zhou 2015	40	40	54.2 ± 9.2	56.5 ± 9.9	Grip the ball hard, hold the ball for 10 s and then relax for 10 s, 25 times/set, 3–5 sets/d.	Grip exercises based on patients willingness	①, ②, ③
Zhou 2021	43	43	53. 28 ± 2.76	53. 57 ± 2.85	Grip the ball to 1/2 of the normal size, hold for 5s, 10–30 times/min, 5 sets/d.	Grip exercises based on patients willingness	①, ②
Zhu 2019	42	42	58. 1 ± 8.6	57.4 ± 7. 8	Hold the ball down by 50% for 10 s, release it for 10 s, 20–30 times, 5-6 groups/d.	Grip exercises based on patients willingness	①, ②, ③, ⑤

① incidence of PICC-related thrombosis; ② maximum venous blood flow; ③ average blood flow; ④ vessel diameter; ⑤ incidence of PICC-related infection rate; ⑥ patients’ satisfaction level; ⑦ blood fibrinogen level.

RCTs = randomized controlled trials.

### 3.3. Quality of included RCTs

**Figure 2.**
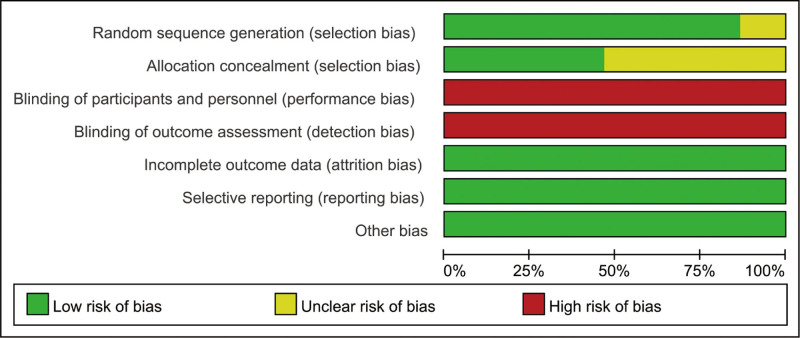
Risk of bias graph.

**Figure 3.**
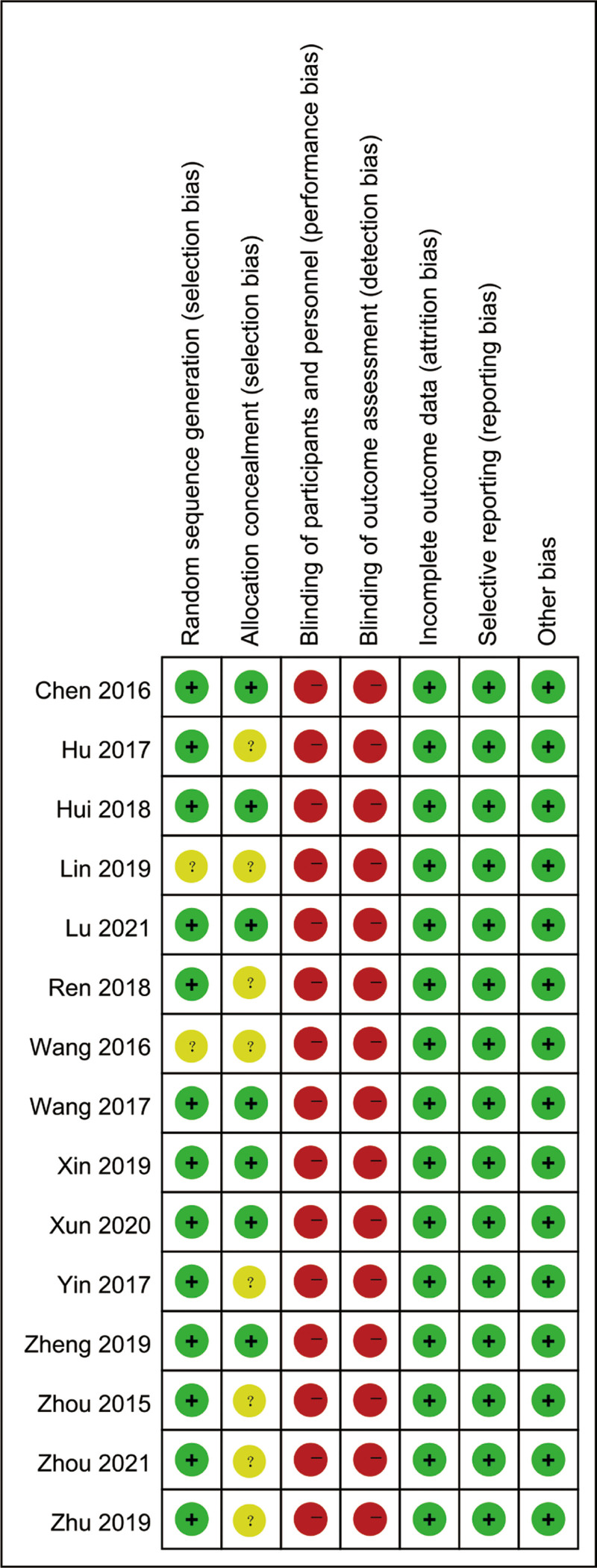
Risk of bias summary.

The quality of included studies are showed in Figures 2 and 3. All of the included 15 RCTs mentioned randomization, 2 RCTs did not describe the methods used to produce a random sequence. And 8 included RCTs did not report allocation blinding. For the blinding of or the personnel and outcome assessment, all included studies did not report the related information. No selective reporting or other significant biases were found.

### 3.4. Synthesized analysis

#### 3.4.1. Incidence of PICC-related thrombosis.

**Figure 4.**
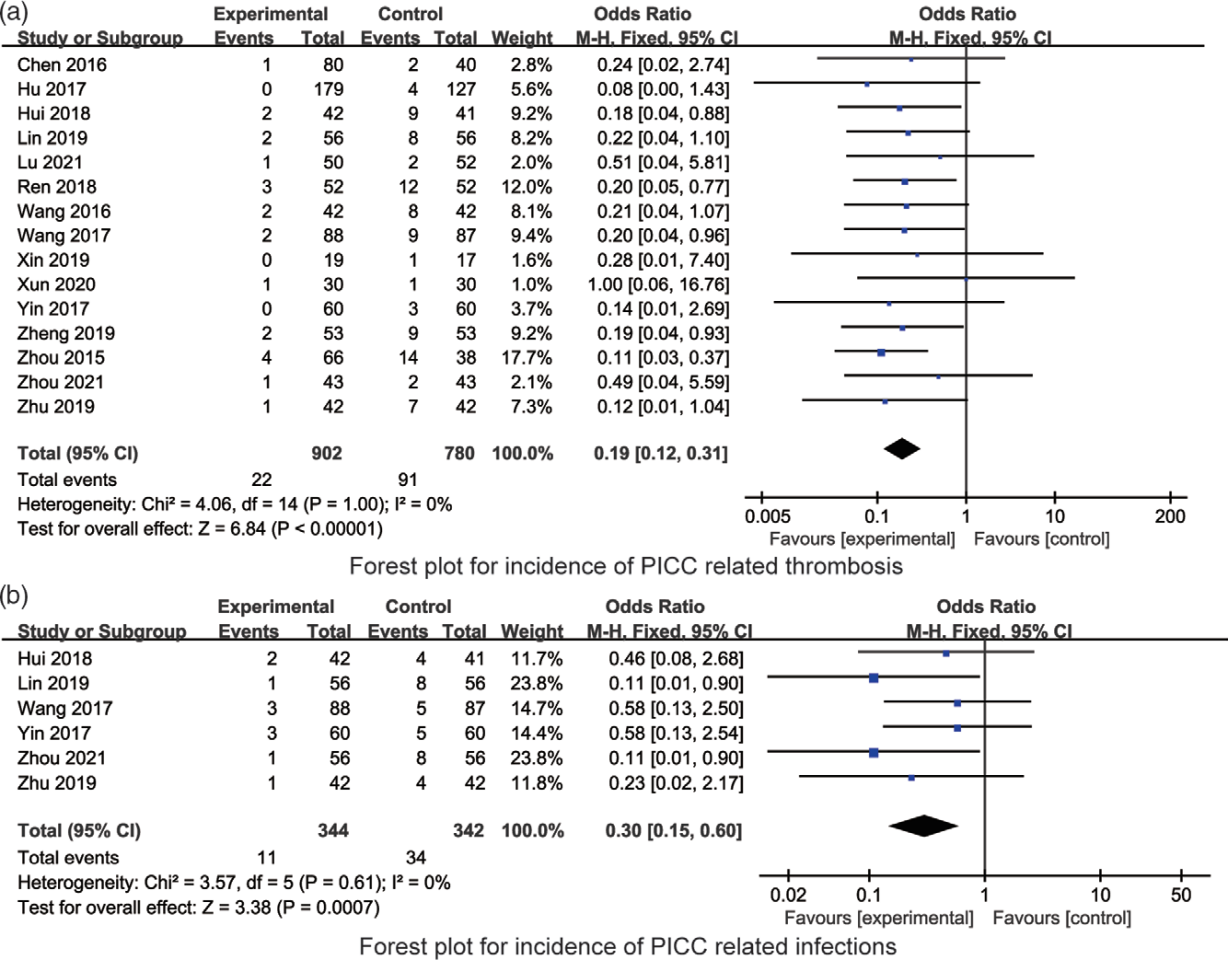
The forest plots for the incidence of PICC-related thrombosis and infections. PICC = peripherally inserted central venous catheter.

All the included 15 RCTs reported the incidence of PICC-related thrombosis. There was no significant heterogeneity in the incidence of PICC-related thrombosis amongst the 15 RCTs (*I*^2^ = 0%, *P* = 1.00), hence fixed model was applied for synthesized analysis. Synthesized result indicated that compared with willful grip exercises, quantified grip exercises were beneficial to reduce the incidence of PICC-related thrombosis in PICC patients (OR = 0.19, 95% CI: 0.12–0.31, *P* < .001, Fig. 4a).

#### 3.4.2. Incidence of PICC-related infections.

All the included 6 RCTs reported the incidence of PICC-related infections. There was no significant heterogeneity in the incidence of PICC-related infections amongst the 6 RCTs (*I*^2^ = 0%, *P* = .61), hence fixed model was applied for synthesized analysis. Synthesized result indicated that compared with willful grip exercises, quantified grip exercises were beneficial to reduce the incidence of PICC-related infections in PICC patients (OR = 0.30, 95% CI: 0.15–0.60, *P* < .001, Fig. 4b).

#### 3.4.3. Maximum venous velocity.

**Figure 5.**
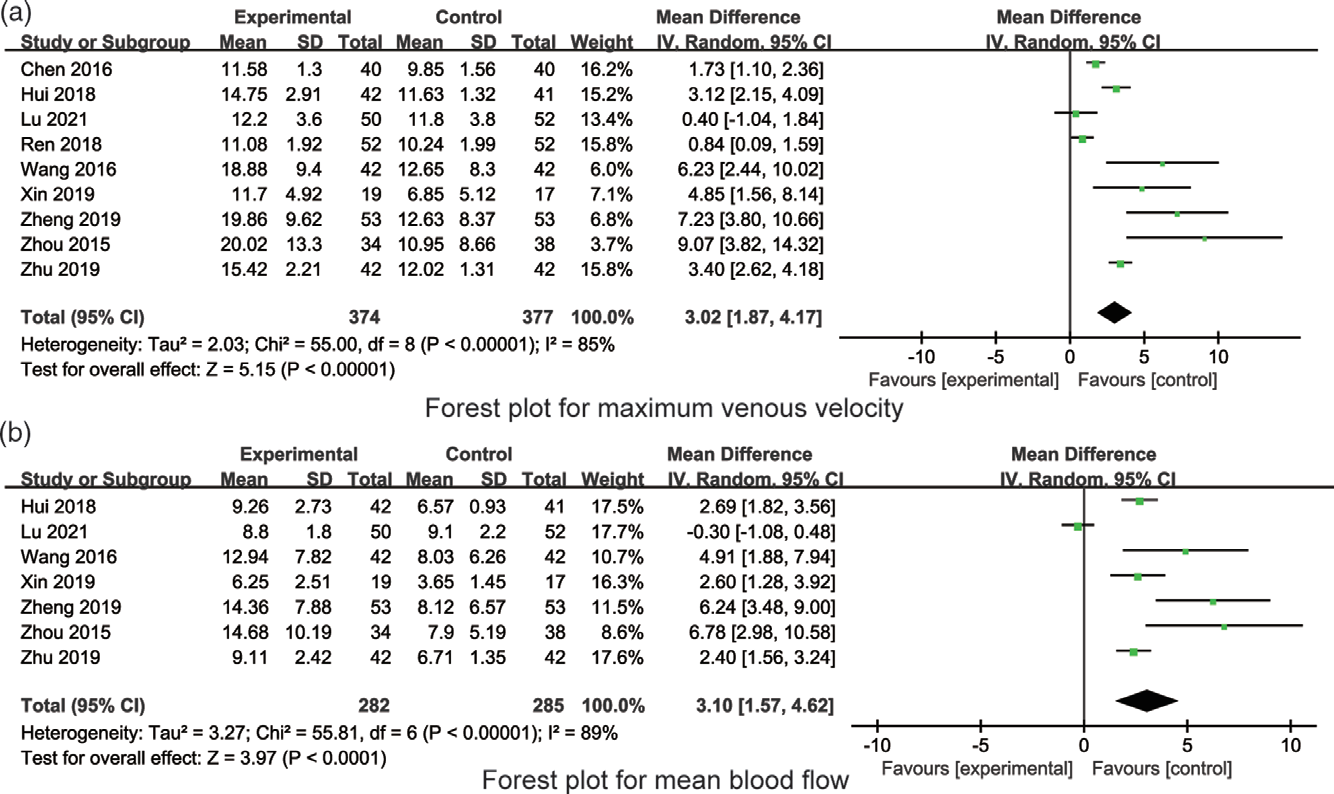
The forest plots for the maximum venous velocity and mean blood flow.

All the included 9 RCTs reported the maximum venous velocity. There was significant heterogeneity in the maximum venous velocity amongst the 9 RCTs (*I*^2^ = 85%, *P* < .001), hence random model was applied for synthesized analysis. Synthesized result indicated that compared with willful grip exercises, quantified grip exercises were beneficial to increase the maximum venous velocity in PICC patients (MD = 3.02, 95% CI: 1.87–4.17, *P* < .001, Fig. 5a).

#### 3.4.4. Mean blood flow.

All the included 7 RCTs reported the mean blood flow. There was significant heterogeneity in the mean blood flow amongst the 7 RCTs (*I*^2^ = 89%, *P* < .001), hence random model was applied for synthesized analysis. Synthesized result indicated that compared with willful grip exercises, quantified grip exercises were beneficial to increase the mean blood flow in PICC patients (MD = 3.10, 95% CI: 1.57–4.62, *P* < .001, Fig. 5b).

### 3.5. Publication biases and sensitivity analyses

**Figure 6.**
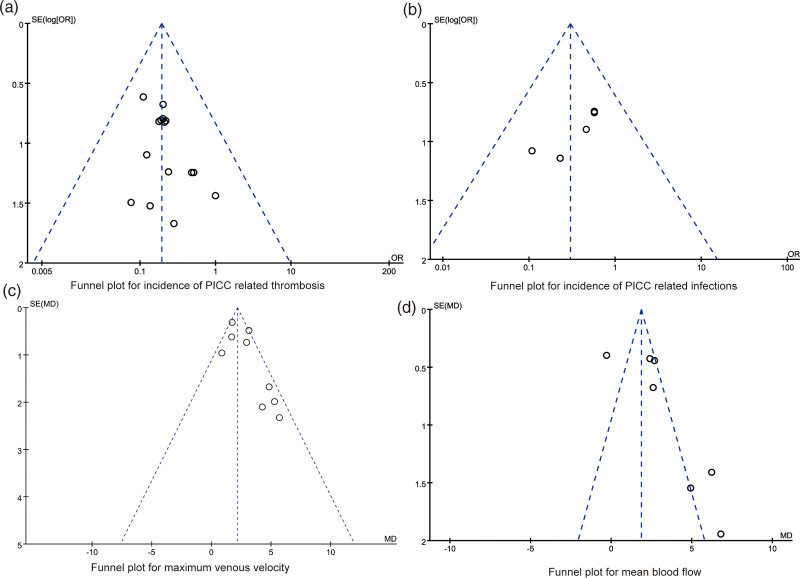
The funnel plots for synthesized outcomes.

As presented in Figure 6, the dots in the funnel plots of synthesized outcomes were evenly distributed, Egger regression test results indicated that there were no publication biases amongst the synthesized outcomes (all *P* > .05).

Sensitivity analyses, which analyze the impact of single 1 study on the overall risk estimate by excluding each study 1 by 1, suggested that the overall risk estimates were not substantially altered by any single 1 study.

## 4. Discussions

PICCs are critical to the treatment and life support of many patients, they aid in the delivery of drugs and blood products, and provide patients with an important vascular access, but long-term indwelling central venous catheters are associated with an increased risk of venous stenosis or thrombosis.^[34,35]^ PICC-related thrombosis can lead to treatment interruption, increased care costs, and may lead to chronic venous occlusion^[36,37]^ and unplanned extubation, postthrombotic syndrome,^[38]^ and, even pulmonary embolism.^[39]^ Some studies^[40,41]^ suggest that the placement of central venous catheters is an independent risk factor for deep vein thrombosis of the upper extremity, and the key reason is that the blood flow velocity in the veins is reduced, resulting in blood stasis. At the same time, long-term catheterization may cause venous stenosis and reduce the diameter of blood vessels, which further affects the blood circulation in the blood vessels.^[42]^ The formation of low-velocity circulation areas and low-shear stress areas may promote the deposition of platelets and fibrinogen, and eventually lead to thrombosis.^[43]^ Some studies^[44,45]^ have found that the venous blood flow rate where the PICC is located can be reduced by as much as 93%, and the magnitude of the reduction in flow rate is positively related to the ratio between vein diameter and catheter diameter. In addition, studies^[46,47]^ have also found that patients with slow deep venous blood flow have an increased risk of thrombosis. In clinical practice, the increased risk of DVT formation is usually attributed to venous blood stasis. Previous studies^[48,49]^ have shown that about 95% of tumor patients generally have a hypercoagulable state, which is often manifested by 1 or more abnormal coagulation function indicators. At the same time, factors such as chemotherapy can easily damage the venous endothelium of patients. After long-term indwelling PICC, venous hemodynamic changes manifested as slowing venous blood flow velocity and increasing the risk of venous thrombosis.

The results of this meta-analysis suggest that compared with willful grip exercises, quantified grip exercises improve the venous hemodynamics and reduces the incidence of PICC-related venous thrombosis and infection in PICC patients. The mechanism of quantified grip exercises^[50]^ in preventing PICC-related thrombosis may be as follows: by isometric muscle contraction, the length of the muscle remains unchanged and the tension changes, the deep flexor digitorum contract sharply, increasing the pressure in the veins of the upper extremities, and the blood flows to the heart instantly. Besides, when the fist is released, the squeezing effect disappears, and making the veins to achieve the purpose of contraction and relaxation to change the blood flow. At the same time, the local venous blood circulation is accelerated, the lymphatic return is promoted, and the incidence of phlebitis infection is reduced. In addition, through regular fisting exercise, the patient’s venous blood is subjected to a certain frequency of pulsed pressure, vascular endothelial cells release vasodilatory factors such as prostacyclin and nitric oxide, which are beneficial to relax vascular smooth muscle and dilate blood vessels. Therefore, during the quantified grip exercises, the inner diameter of the blood vessel increases to a certain extent, which is beneficial to the venous blood circulation, reduce the risk of infection.^[25]^ Besides, quantified grip exercises are easy to learn and operate with few adverse reactions, and patients are highly satisfied and can persist in exercising.^[27,29]^

At present, the mode of functional exercise after PICC has not been unified in clinical practice. The most common functional exercise after PICC is grip exercise, but there is no unified standard for the strength, frequency and duration of grip. Some studies^[51,52]^ have found that active fisting can effectively prevent the formation of PICC-related upper extremity venous thrombosis and improve hemodynamics. However, the effect of grip exercise is not positively correlated with strength, frequency and length. Previous study^[53]^ has found that 30 times/minute of grip exercise can effectively improve venous blood flow, which is the best frequency of grip exercise after PICC. In addition, a study^[54]^ has found that the patient’s use of the grip ball in the palm of the hand can give the patient a sense of reality, enhance the afterload of the upper arm muscle group contraction, and effectively promote blood circulation. At the same time, some studies^[55,56]^ believe that the use of electronic grips to guide patients to make fist exercises can enhance the effect of grip exercises. When the frequency of electronic grips is 25 times/minute and the exercise duration is 2 minutes, the blood flow and speed of patients can reach the maximum, this maximizes patient benefit.^[57,58]^

There are certain limitations of this study that deserve to be considered. First, the RCTs included in this meta-analysis are all Chinese literatures, and no RCT reports from other countries have been found so far, and the research results may have certain population and geographical biases. Second, none of the studies included in this meta-analysis reported the blinded design of the study and outcome measures, and the quality of the RCTs was not high. Third, there are still some differences in the method and intensity of quantified grip exercises in the included studies, which still needs to be further improved by subsequent multi-regional, high-quality, and large-sample studies.

## 5. Conclusions

In conclusion, the results of this meta-analysis have showed that compared with willful grip exercises, quantified grip exercises can reduce the incidence of PICC-related thrombosis and infection, and improve the venous blood circulation in PICC patients. The results of this study can provide a reference for the construction of evidence-based guidelines for the prevention of complications such as PICC-related thrombosis, and provide a scientific basis for clinical medical workers to guide patients with PICC catheters to perform hand exercises. It is worth noting that, there is no uniform standard for quantifying the tensity, duration and frequency of fisting exercises, high-quality, large-sample, multicenter RCTs are still needed to further explore the effects and safety of quantified grip exercises in PICC patients, to improve the prognosis of PICC patients.

## Author contributions

**Investigation:** Hongliang Luo, Cheng Jin, Xiaohong Li, Yinzhu Jiang, Jing Zhou.

**Formal analysis:** Jing Zhou.

**Visualization:** Jing Zhou.

**Writing – original draft:** Jing Zhou.

**Writing – review & editing:** Jing Zhou.
